# Surface Enhanced Raman Spectroscopy for Single Molecule Protein Detection

**DOI:** 10.1038/s41598-019-48650-y

**Published:** 2019-08-26

**Authors:** Lamyaa M. Almehmadi, Stephanie M. Curley, Natalya A. Tokranova, Scott A. Tenenbaum, Igor K. Lednev

**Affiliations:** 1 Department of Chemistry, University at Albany, SUNY 1400 Washington Avenue, Albany, NY 12222 USA; 2 College of Nanoscale Science and Engineering, SUNY Polytechnic Institute 257 Fuller Road, Albany, NY 12203 USA; 3 The RNA Institute, College of Arts and Science, University at Albany, SUNY, 1400 Washington Avenue, Albany, NY 12222 USA

**Keywords:** Bioanalytical chemistry, Techniques and instrumentation

## Abstract

A two-step process of protein detection at a single molecule level using SERS was developed as a proof-of-concept platform for medical diagnostics. First, a protein molecule was bound to a linker in the bulk solution and then this adduct was chemically reacted with the SERS substrate. Traut’s Reagent (TR) was used to thiolate Bovine serum albumin (BSA) in solution followed by chemical cross linking to a gold surface through a sulfhydryl group. A Glycine-TR adduct was used as a control sample to identify the protein contribution to the SER spectra. Gold SERS substrates were manufactured by electrochemical deposition. Solutions at an ultralow concentration were used for attaching the TR adducts to the SERS substrate. Samples showed the typical behavior of a single molecule SERS including spectral fluctuations, blinking and Raman signal being generated from only selected points on the substrate. The fluctuating SER spectra were examined using Principle Component Analysis. This unsupervised statistics allowed for the selecting of spectral contribution from protein moiety indicating that the method was capable of detecting a single protein molecule. Thus we have demonstrated, that the developed two-step methodology has the potential as a new platform for medical diagnostics.

## Introduction

Accurate diagnosis of human disease often requires time-consuming, expensive biomedical testing, and the involvement of highly trained personnel. This is partly because relatively simple bioanalytical tests typically suffer from low sensitivity and selectivity and require large sample volumes for the analysis^1,2^. This is particularly true for the detection of RNAs, which are used as disease diagnostic biomarkers^3^ in urine and serum samples, and is predominantly limited to traditional nucleic acid based quantification techniques. These methods typically involve microarray and qPCR analysis that often require time-consuming amplification steps and skilled personnel, which can limit their utility for the clinical setting or as a point-of-care diagnostic^4^. Surface enhanced Raman spectroscopy (SERS) has advanced significantly and now has the potential to overcome these limitations while maintaining its high sensitivity. SERS has proven its ability to detect a single molecule deposited on colloidal metal or a substrate surface^5^ and offers high specificity^6^. In SERS, the Raman signal of adsorbed molecules is amplified at “hot spots” on the substrates surface^7^. Typically, when SERS substrates are used for disease diagnostics, a surface is functionalized with an antigen to provide the necessary selectivity to a specific biomarker^8,9^.

Tenenbaum and co-workers^10^ have recently developed a novel approach for biomedical diagnostics based on the binding of an engineered RNA molecule to a specific targeted microRNA molecule that ‘switches’ the structural conformation of the engineered RNA strand upon binding^10^. The presence and subsequent binding of the targeted microRNA to the engineered RNA creates a structurally interacting RNA (sxRNA) three-way junction with a central stem-loop, which can be used to recruit the binding of an RNA-binding protein (RBP). The selective capture of a target protein is a central component of this sxRNA technology and directly correlates to the amount of targeted microRNA present. We speculated that surface enhanced Raman spectroscopy (SERS) might be an ideal technique for detecting the sxRNA bound protein with a high degree of sensitivity and specificity. With this long-term goal in mind, we first developed the two-step process described here for the simple detection of a protein at the single molecule level using SERS.

Using our approach, a protein is first bound to a linker in a bulk solution and then the formed adduct is chemically reacted with the SERS substrate. Bovine serum albumin (BSA) was chosen as a proof-of-concept protein for this study and has been used before for SERS detection at a single molecule level^11,12^. Traut’s reagent (TR) was chosen as a protein linker to the gold SERS substrate. TR reacts spontaneously with primary amine groups. This ring-opening reaction results in the formation of a free thiol group. Since BSA structure is rich with lysine amino acid residues^13^ containing free primary amine groups, it readily reacts with the TR molecules. As a result, the protein-linker adduct can potentially contain multiple free thiol groups to be covalently attached to the SERS gold substrate in the second step. Other amino acid residues, such as asparagine and glutamine, contain amide groups in their side chains and have limited reactivity with TR.

In this study we examine the potential of protein detection at a single molecule level when small linker molecules react with a protein in bulk solution followed by attachment to the SERS substrate. This two-step process is followed by acquiring Raman spectra from multiple points on the substrate via automatic mapping. Fluctuations of consecutive spectra measurement at individual spots were also analyzed. A statistical model was developed to support the results of differentiating the protein contribution in protein-linker SER spectra. Principle Component Analysis (PCA) is used for the statistical analysis, which is a multivariate analytical method used to highlight spectroscopic variations via computing principal components (PCs)^14^.

SERS at a single-molecule level is usually accompanied by the fluctuations of Raman signal^15^. The term “fluctuations” describes several spectral behaviors, such as frequency shifts (spectral wandering), on-off cycling (blinking), and peak intensity fluctuations^16^. Blinking is defined as the alternation between disappearance of Raman signal in the off-mode and appearance of Raman signal in the on-mode^15^. In this article, the term “fluctuations” is used as described earlier, and the term “blinking” will be used to specifically indicate the appearance and disappearance of Raman signal. Although, the term “blinking” is used often in literature, the blinking phenomenon is still not well-understood. Signal appearance and disappearance can be a result of the molecule movement or changes in the hot spot geometry, movement of colloidal particles in particular. We refer the reader to the comprehensive review article^17^ for more information. As fluctuations of Raman signal is expected, collecting multiple spectra provides rich information about covalently bound molecules. Two typical approaches are used for generating a SER spectral dataset, (i) acquiring spectra from multiple spots on a SERS substrate using automatic mapping for example and (ii) recording multiple consecutive spectra from individual substrate spots^18,19^.

## Results and Discussion

### Mapping of BSA-TR adduct on SERS substrate

**Figure 1:**
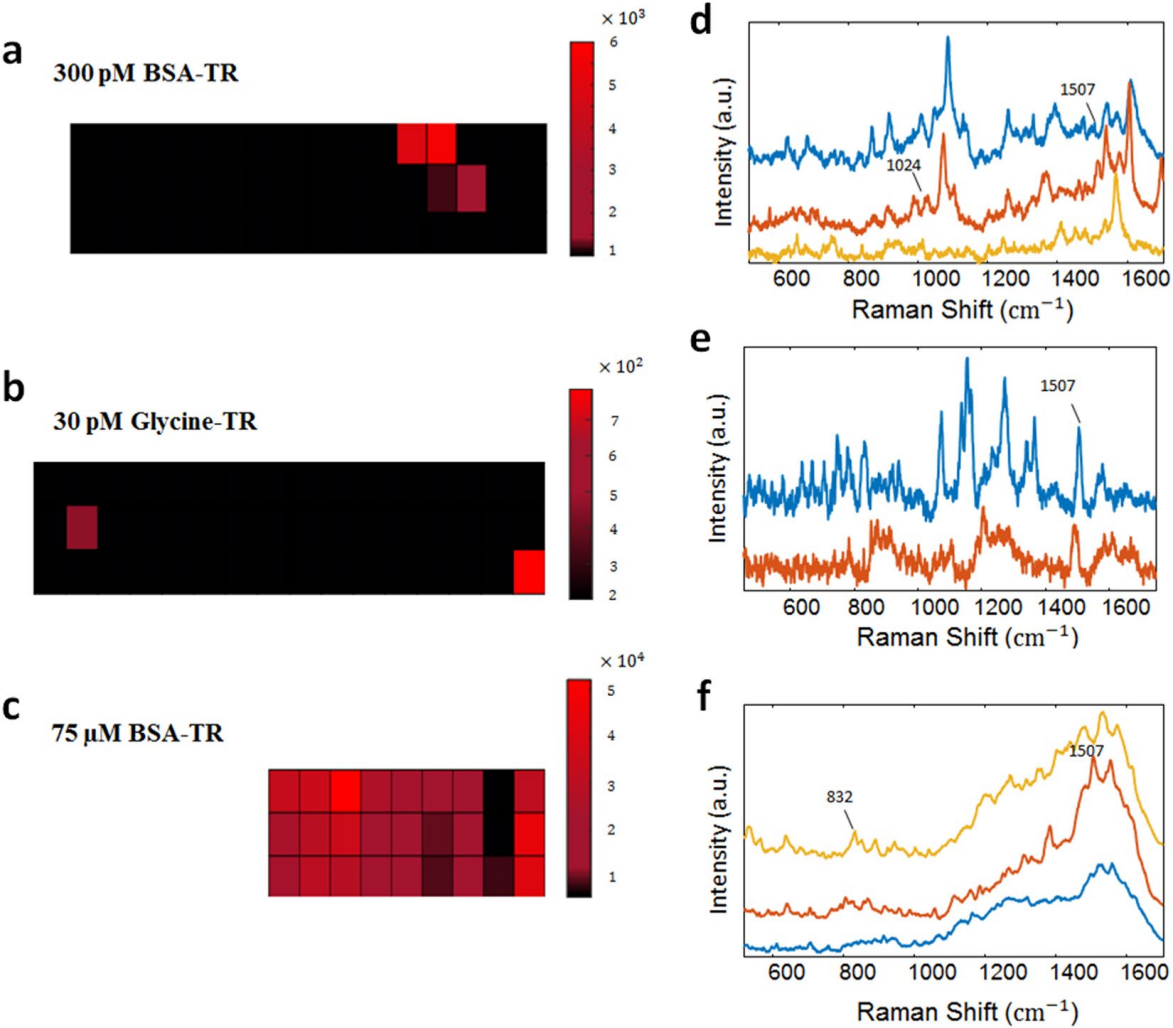
Spectroscopic maps for substrates with a low loadings of BSA-TR adduct (**a**), and Glycine-TR adduct (**b**) and high loading of BSA-TR adduct (**c**) built using the intensity of Raman signal at 1532, 1501 and 1523 cm^−1^, respectively. Representative SER spectra of BSA-TR adduct deposited at low concentration (**d**), and Glycine-TR adduct deposited at low concentration (**e**), and high concentration of BSA-TR adduct (**f**). The mapping areas for a, b and c are 80 × 15 µm, 85 × 15 µm, and 45 × 15 µm, respectively.

It is well accepted in the field that a SERS signal is generated by a hot spot occupied by an analyte. If the amount of an analyte on the SERS substrate is low, selected spots only generate Raman signal. Should such situation be observed using the automatic mapping approach, it is indicative of SER spectra generated from a single hot spot^20^. For comparison, we collected SER spectra from substrates loaded with different amounts of BSA-TR adduct. The automatic mapping was conducted with 5-µm step size. It is expected that both BSA and TR moiety of the adduct contribute to the spectra. The results of the automatic mapping of SERS substrates obtained due to the exposure to BSA-TR solutions at 300-pM and 75-µM concentrations are shown as spectroscopic maps in Fig. 1(a,c). Mapping the substrate with a high BSA-TR loading often showed noticeable Raman spectra, although with some spectral variation. In contrast, mapping the substrate with a low BSA-TR loading shows only a few spots that produce SER spectra. Similar behavior was found when a substrate loaded with 30-pM Glycine-TR solution was subjected to spectroscopic mapping (Fig. 1b). This is an expected trend since the low number of deposited molecules on the SERS substrate decreases the probability for a hotspot to be occupied^20^. Representative spectra obtained by mapping substrates with high and low loadings of BSA-TR and low loading of Glycine-TR are shown in Fig. 1(d–f). In all of these cases, significant spectral variations are evident from spot to spot. Despite these variations, SER spectra obtained from substrates with high and low loadings of BSA-TR could be easily differentiated by visual inspection. SER spectra obtained from the substrate with a high BSA-TR loading exhibit relatively broad peaks that could be tentatively explained by the contribution from several hotspots to each individual spectrum. Singhal *et al*.^21^ has reported similar behavior of SER spectra of a small photoactive yellow protein, where both high and low solution concentrations were used. The spectra of the protein showed broad peaks at the high concentration that was assigned to the case of mutli-molecule SERS. While the protein spectra obtained at the low concentration exhibit narrow peaks and have been assigned to single protein molecule spectra. More detailed description of BSA-TR behavior on the SERS substrate could be complicated by the protein denaturation due to the effect of gold surface^22^ and hydrophobic interaction with the protein in particular^12^.

### Consecutive spectral measurements from an individual spot on SERS substrate

**Figure 2:**
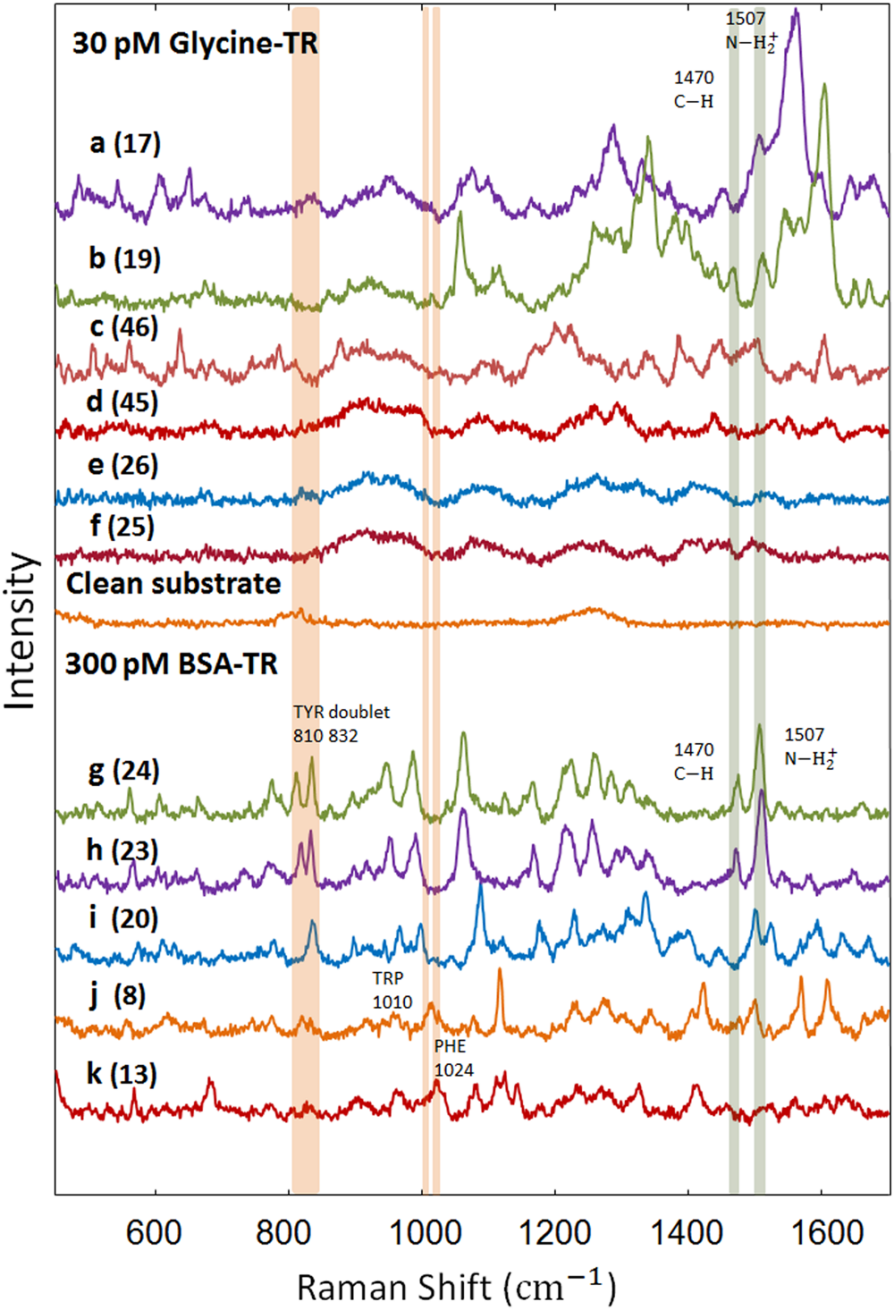
Representative spectra of Glycine-TR (**a**–**f**) and BSA-TR (**g**–**k**) and that were collected from individual spots on a corresponding substrate. The highlighted bands (in orange) show the contribution of the protein to BSA-TR spectra. Bands highlighted with green show the contribution of TR to both BSA-TR and Glycine-TR spectra. Spectra e and f show the blinking behavior of Glycine-TR spectra. A representative clean substrate spectrum shows no Raman peaks. All spectra are shown at the same Y-scale. Numbers in brackets represent individual spectra consecutive numbers from Fig. 3.

Consecutive multiple spectra were collected from individual spots on substrates with a low loading of BSA-TR and Glycine-TR. Representative spectra of the two substrates are shown in Fig. 2. Both series of spectra acquired form individual spots of substrates loaded with Glycine-TR and BSA-TR show significant spectral fluctuations and each individual Raman peak in these spectra show significant variations with spectrum number. Supplementary Fig. S1a,b show the intensity change for selected peaks across 48 spectra of the low loadings of BSA-TR and Glycine-TR. In addition to spectral fluctuations, the low loading Glycine-TR spectra also show blinking. Specifically, spectra e and f in Fig. 2 clearly show the disappearance of Raman peaks that can be attributed to the blinking behavior^15^. It is interesting that no spectra lacking Raman peaks were found in the case of low loading BSA-TR sample that will be further discussed below.

**Figure 3:**
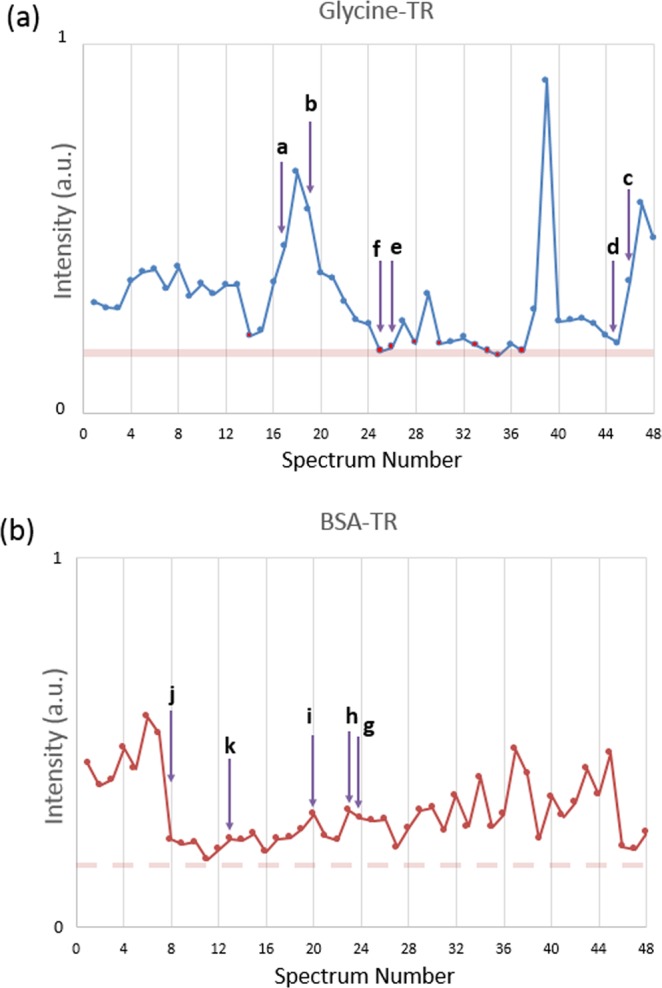
The area under a spectrum curve of the low loading of Glycine-TR (**a**) and BSA-TR (**b**) versus the consecutive spectrum number. Red points in plot (**a**) represent apparent blinking when no Raman spectral features are noticeable. Plot (**b**) shows spectral intensity fluctuations while all spectra exhibit evident Raman scattering lines. The dotted line represents the level of expected blinking (background signal only without Raman scattering lines) that has the same intensity level as the pink line in plot (**a**). The letters correspond to the spectra presented in Fig. 2.

**Figure 4:**
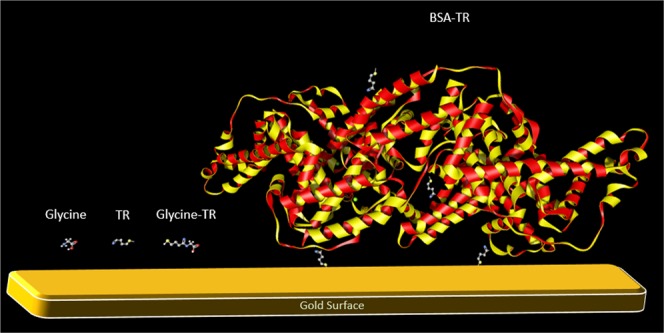
Comparison of relative sizes of Glycine, reactive TR, Glycine-TR and BSA-TR.

The area of each spectrum obtained for the low loading BSA-TR and Glycine-TR is plotted in Fig. 3(a,b) vs. the spectrum number. The variation of the area shows the change in the overall Raman signal intensity from spectrum to spectrum. In Fig. 3(a), the low loading Glycine-TR shows apparent blinking by some spectra (corresponding points are colored in red). In the low loading BSA-TR spectra, although spectra change significantly, only spectral fluctuations are observed without apparent blinking as indicated above (Fig. 3b). A pink dotted line shows expected area range of blinking when relatively compared to the low loading Glycine-TR blinking spectra. The no-blinking observation of the low loading of BSA-TR spectra can be explained by the relatively large size of BSA molecule (Fig. 4) in comparison to Glycine-TR. The latter (large size) might limit the movement of BSA molecule with respect to a hot spot. Alternatively, a single BSA molecule could potentially interact with two or more hot spots and consequently reduces significantly the probability of blinking while the acquired individual SER spectrum consists of signals from several hot spots. Spectral contribution from more than one hot spot could also explain an apparent more frequent appearance of individual Raman peaks in spectra recorded from individual spot on the low loading BSA-TR substrate (See Supplementary Fig. S1b) relative to those obtained for the low loading Glycine-TR (See Supplementary Fig. S1a). Should the latter be the case, the number of hot spots, which a single BSA molecule interacts with, should be small, so no spectral broadening would occur as in the case of SERS of substrate with high BSA-TR loading (Fig. 1f). A BSA molecule is expected to have an interaction with a few hot spots because TR can potentially react with 30–35 lysine residues of BSA^23^. However, the result of Ellman’s assay quantification of thiolated residues showed that about 10% of the primary amine groups on BSA were modified. If a BSA molecule is linked to the substrate by more than one TR group, it is expected that its mobility would be restricted.

Although the spectra of low loadings of Glycine-TR and BSA-TR fluctuate, some tentative peak assignments can be made. Raman peaks, which are present in the low loading BSA-TR spectra and are absent in those of Glycine-TR could be assigned to protein vibrational modes. Specifically, the peaks at 1010 cm^−1^ in spectrum (j, Fig. 2) and 1024 cm^−1^ in spectrum (k) are tentatively assigned to the indole asymmetric ring breathing mode of tryptophan^24^ and C-H in-plane bending mode of phenylalanine^25,26^, respectively. The peaks at 810 cm^−1^ in spectra (g,h,j) and 832 cm^−1^ in spectra (g–j) are tentatively assigned to tyrosine Fermi resonance doublet^27^. The reason of detecting some of the protein’s assigned peaks in different spectra could be due to continuous motion of the molecule where only the region that is in close proximity to the surface is enhanced^28^. Although some peaks in BSA-TR spectra could sometimes be solely a result of a protein vibration, the possibility of overlapping with TR peaks complicates their assignment. Thus, only peaks that solely appear in BSA-TR spectra and not in Glycine-TR spectra are considered to be due to protein contribution. These peaks are tentatively assigned to specific amino acid sidechains as indicated in Table 1.

**Table 1:** Tentative assignment of Raman peaks in BSA-TR and Glycine-TR SER spectra resulting from protein (BSA) and linker (TR) parts.

Vibrational mode	SERS Peaks (cm^−1^)	References
Tyr doublet (BSA)	810, 832	^27^
Trp w16 (indole asymmetric ring breathing mode) (BSA)	1010	^24^
Phe v18a (C-H in-plane bending mode) (BSA)	1024	^25, 26^
C-H (TR)	1470	^32^
${{\rm{NH}}}_{2}^{+}$ (TR)	1507	^31^

### Tyrosine Raman peaks

SERS has been previously used to study Bovine serum albumin (BSA)^29^ including that at a single molecule level^11,12^. As a result, extensive information about BSA SER spectra can be found in the literature. Specifically, a SERS study has reported that BSA experiences certain conformational changes as it interacts with a gold surface^22^. The highlighted 810/832 cm^−1^ peaks in Fig. 2 are tentatively assigned to tyrosine Fermi resonance doublet^27^. Both peaks of this doublet are found in BSA-TR spectral dataset only. As shown in Fig. 2(g–j), the tyrosine Fermi doublet exhibits an interesting behavior in the BSA-TR SER spectra. In some spectra, only one peak is present at 832 cm^−1^ which is tentatively assigned to the out-of-plane deformation^12^. Brulé.T *et al*.^12^, have reported a similar behavior of tyrosine Fermi doublet peaks. Their BSA SER spectra sometimes show only one of the doublet peaks that corresponds to in-plane or out-of-plane vibrations^12^. The breaking down of the doublet is explained to be due to the structural changes of BSA as a result of interaction with the substrate surface^12^. This is reasoned by the fact that gold is hydrophobic and preferably interacts with other hydrophobic amino acid residues^12^. This type of interaction induces structural changes that constrains the orientation of tyrosine^12^. In other words, one of the peaks only appeared in an individual Raman spectrum that was assigned to the effect of metal surface on the adsorbed molecule^12^. This specific behavior of protein-metal interaction in SERS supports further our conclusion about the protein detection. This conclusion is further supported by the fact that a BSA molecule contains 21 tyrosine residues^30^, and many of them are close to the protein surface^12^ which makes the probability of a tyrosine residue to be in a close proximity to the gold surface higher.

Since TR is immediately connected to the substrate, it should always contribute to SER spectra. The other highlighted peaks in Fig. 2 are detected in both low loadings of Glycine-TR and BSA-TR spectra and can be tentatively assigned to TR. Specifically, the peak at 1507 cm^−1^, which is tentatively assigned to ${{\rm{NH}}}_{2}^{+}$ ^31^, is found in spectra a–c of the low loading Glycine-TR, and in spectra g and h of the low loading BSA-TR. The peak at 1470 cm^−1^ is tentatively assigned to C-H^32^. This peak is detected in the low loadings spectra g and h in BSA-TR, and in the low loading spectra of b in Glycine-TR.

The low loading Glycine-TR sample was primarily used as a control to get its SER spectroscopic signature and evaluate its potential contribution to BSA-TR spectra. Considering that SERS is very sensitive to the molecular structure and TR changes its cyclic conformation to aliphatic upon binding to a primary amine group, it was necessary to use a molecule containing a primary amine group for the control sample. Glycine was chosen to be attached to TR to simulate TR moiety in BSA-TR. Glycine residue contains a carboxyl group, and it has been reported that glycine SER spectra on gold substrates enhance only *v*(C–COOH) and *v*_*as*_(COOH) modes at 917 cm^−1^ and 1596 cm^−1^ ^25^, respectively. As a consequence, the assigned protein contribution peaks are not likely to be a result of the glycine spectral contribution. In fact, the identified protein peaks are more likely to be from aromatic amino acids that have both large cross section^11^ and higher affinity to react with gold^12^.

### Statistical analysis of spectral data

**Figure 5:**
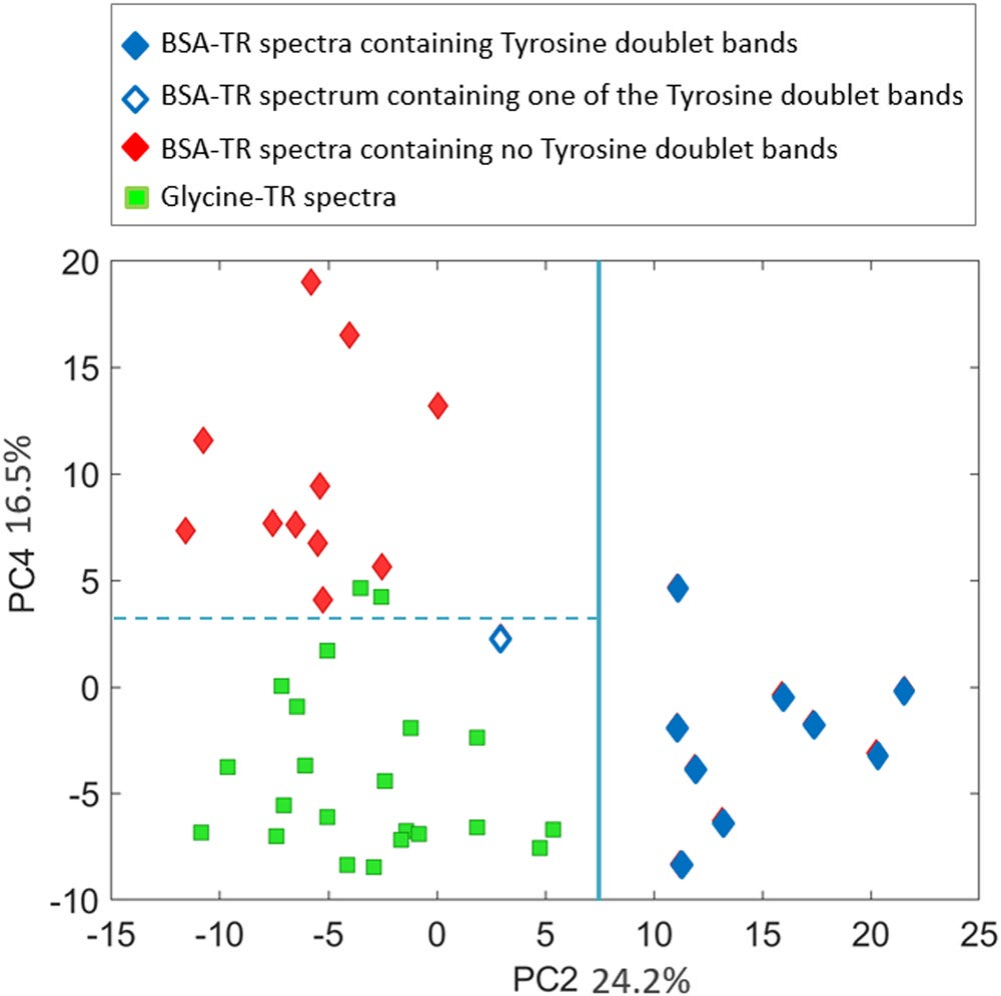
A PCA score plot shows clear differentiation of BSA-TR and Glycine-TR moieties based on multiple consecutive SER spectra acquired from individual active spots. PC2 differentiate clearly individual Glycine-TR spectra and BSA-TR spectra containing one or two Tyrosine Raman bands. PC4 separates individual Glycine-TR spectra and BSA-TR spectra containing no Tyrosine Raman bands (see text for more details).

Since the spectra of both BSA-TR and Glycine-TR fluctuate, visual analysis to differentiate BSA-TR dataset from Glycine-TR dataset is complicated. For this reason, we utilized statistical analysis to differentiate Raman spectra obtained from these two substrates. Specifically, we used unsupervised method, principle component analysis (PCA) to explore the obtained spectral datasets. We restricted principle components to non-negative values to simplify their interpretation. Figure 5 shows a score plot based on two components PC2 and PC4. Together, these two components cover 40.7% of total variance. The spectrum index of each dataset with respect to individual PCs shows that PC2, and PC4 have a good level of variation between the low loading BSA-TR and Glycine-TR spectra (See Supplementary Fig. S2). The majority of spectra in each dataset are in an opposite trend with respect to the other dataset. Figure 5 shows that principle component 2 (PC2) allows for a complete differentiation of BSA-TR spectra containing tyrosine Raman bands from all Raman spectra of Glycine-TR adduct. PC2 is the only principle component which exhibits these spectral bands (See Supplementary Fig. S3). The rest of BSA-TR Raman spectra could also be differentiated from the Glycine-TR spectra based on PC4, although it is not obvious what Raman bands allow for this differentiation.

Our results suggest that the detection of BSA-TR adduct is at the single molecule level. The observed spectral fluctuations are a well-known characteristic of single-molecule detection in SERS^16^. This behavior can be explained by the molecule movement with respect to the hot spot and the intense plasmon field specifically^15^. Spectral fluctuation alone does not necessarily prove a single molecule behavior^33^. Although blinking is not observed in BSA-TR spectral dataset due to the discussed reasons, the detection of single protein molecule is further supported by the mapping results shown in Fig. 1. The observed heterogeneous distribution of Raman signal of the low loading samples indicates that only a few hot spots on the substrate are occupied, and as such it should be occupied by a single molecule.

In conclusion, the two-step SERS approach we used here has the ability to detect a single protein molecule with a high specificity, which makes it a promising tool for many applications including disease diagnostics including those targeting microRNA such as the sxRNA approach described earlier. The goal of this study was to investigate the potential of SERS to detect a protein at the single molecule level through the formation of a covalent bond between SERS substrate and protein-linker adduct. SERS substrates were manufactured using an electrochemical deposition of gold. Raman spectra were collected using the automatic mapping followed by multiple consecutive spectral measurements at selected spots. Since BSA has been extensively studied due to its abundance and high stability, and TR can act as a covalent cross-linker between BSA and the gold substrate, BSA-TR adduct was chosen for this study. Raman spectra of BSA-TR adduct on the SERS substrate have shown a contribution of both the protein (BSA) and the small molecule linker (TR) when deposited from a solution with an ultralow concentration of 300 pM. In a control experiment, glycine was attached to TR instead of the protein to initiate the attachment to the gold substrate.

Since SER single molecule spectra vary significantly with the position on the substrate and during the recording from an individual spot, no single spectrum makes a complete representation of a specific species. A statistical model for separating BSA-TR and Glycine-TR adducts was developed using the positive principle component loadings for the PCA analysis of SER spectral datasets. The results of the statistical analysis clearly showed a differentiation between the two classes.

As the spectra of Glycine-TR and BSA-TR were compared both visually and statistically, it was possible to identify Raman peaks that were present in BSA-TR spectra and not in Glycine-TR spectra, and consequently can be assigned to protein vibrational modes. In particular, tyrosine Fermi doublet peaks at 810/832 cm^−1^ were detected in several BSA-TR spectra unlike Glycine-TR spectra, which lack these peaks. Other peaks at 1010 cm^−1^ and 1024 cm^−1^ were assigned tentatively to tryptophan and phenylalanine residues, respectively, based on literature data. The obtained results indicated single-molecule detection of low loading of BSA-TR on the SERS substrate. That was evident from the known single molecule characteristic of SERS, spectral fluctuation, and is supported by the heterogeneity of spectral maps at the ultralow concentration of BSA-TR loading. Due to its high sensitivity, ability to detect at the single molecule level, and flexibility for conjugation of other biological molecules, the developed two-step methodology shows great potential as a new platform for medical diagnostics. We plan to further validate SERS as a sensing platform for nucleic acid and RNA-binding protein detection, including using the microRNA-sxRNA approach in future studies.

## Methods

### Materials

Traut’s reagent (2-iminothiolane) was obtained from Toronto Research Chemicals (Ontario, Canada). Bovine Serum Albumin (BSA), Ellman’s reagent, and glycine were purchased from Sigma Aldrich. The PD-10 Desalting Column (Sephadex G-25 M) was purchased from GE Healthcare, and the Vivaspin 500 concentrators were purchased from Sartorius. Gold-containing solution was obtained from Transene Company, Inc.

### BSA-TR and glycine-TR conjugation

BSA was reacted with Traut’s reagent at a 1:50 molar ratio in PBS for one hour at room temperature. BSA-TR adducts were purified using a PD-10 Desalting Column (5 kDa MWCO) and then concentrated using a Vivaspin 500 concentrator with a 30 kDa MWCO. The obtained BSA protein content was determined using a Nanodrop spectrophotometer (ND1000, ThermoFisher Scientific) and BSA molar absorption coefficient ε_280_ = 43,820 M^−1^cm^−1^ ^34^. After purification and quantification, BSA-TR was used for conjugation to gold.

Glycine was reacted with Traut’s reagent at a 1:1 molar ratio in PBS for one hour at room temperature. The obtained solution was used for reaction with a gold substrate. Ellman’s assay was used to quantify the number of TR conjugated to BSA and glycine. Each modified molecule was reacted with an excess of Ellman’s reagent and the sample absorbance was measured at 412 nm. The number of free thiol groups added was quantified using the below Eq. (1):

1 $$\frac{\mathrm{Free}\,\,{\rm{thiols}}}{{\rm{Molecule}}}=\frac{{\rm{\Delta }}A}{{{\rm{\varepsilon }}}_{ER}\times {\rm{M}}}$$

where ΔA represents the difference between the absorbance values at 412 nm before and after addition of Ellman’s reagent, ε_ER_ represents the extinction coefficient of Ellman’s reagent at 412 nm (14150 M^−1^cm^−1^, as shown by Collier^35^), and M represents the molarity of BSA in solution.

### SERS

SERS substrates were prepared by electrodeposition of gold at room temperature from the cyanide solution “Pure Gold SG-10” on a silicon substrate coated with a layer of Cr/Au. The deposition of gold was through applying constant potential of 4.9 V to an area of 1 cm^2^ of the substrates for 30 s. After the electrodeposition process, the substrates were washed with Nano-purified DI water and ethanol. Raman spectra were measured at random spots of the substrate surface to check for potential contaminations. Substrates, which showed no contamination, were used for SERS experiments.

15 µM of BSA-TR was diluted to 300 pM and 17 mM of Glycine-TR was diluted to 30 pM before reaction with the SERS substrate to achieve a “low loading” of the analyte attached to the substrate. Most probably the binding efficiency of TR to the gold surface in BSA-TR adduct is lower than that in the case of Glycine-TR due to steric effects. In other words, TR can bind to various parts of the protein and some TR groups could not have access to the gold surface as illustrated in Fig. 4. As the binding efficiency of BSA-TR adduct is expected to be lower than that of Glycine-TR adduct, the solution concentration of BSA-TR adduct used for the deposition is higher than that of the Glycine-TR. Although BSA-TR adduct is used at the higher concentration, it is kept within pM range in order to form only a few active hotspots on the SERS substrate area subjected to the automatic mapping. A higher concentration of 75 µM of BSA-TR was used to achieve a “high loading” of the BSA-TR adduct on the SERS substrate and demonstrate the activity of the substrate in all points on the surface. 3 µL of each solution were deposited on the gold substrates and let to dry for two hours after which the substrates were washed with PBS buffer solution to remove any unattached material. It has been reported that 1 hour of incubation is sufficient for TR to bind to gold surface^36^.

Raman spectra were acquired using a Renishaw inVia Raman microscope with a 20x objective. 785-nm laser light was used for the excitation at 55-mW power with an accumulation time of 10 s. SER spectra of BSA-TR and Glycine-TR were collected via automatic mapping with a 5-µm step size. A low numerical aperture objective (20x) was used to cover a large SERS substrate area during the mapping procedure and kept for all other SERS measurements for consistency. Multiple consecutive spectra were also measured from individual spots for each sample.

21 spectra of BSA-TR and 22 spectra of Glycine-TR were analyzed using R-project software version 3.4.4. A fingerprint spectral region for the analysis was between 1700 cm^−1^ and 450 cm^−1^. The “nsprcomp” package^37^ was used to constrain the PCA to non-negative loadings only. The goal of statistical analysis was to differentiate two SERS datasets obtained for BSA-TR and Glycine-TR using physically meaningful components (positive values only). SER spectra were baseline corrected using automatic weighted least squares (6^th^ -order polynomial) and normalized using standard normal variate method to reduce the variance within each dataset.

A schematic representation of relative sizes between BSA, Glycine, and TR in Fig. 4 were obtained using ChemDoodle 3D V.3.0. Structural models of BSA and glycine were downloaded from PDB (3V03) and PubChem (CID 750), respectively. Glycine-TR and TR were drawn utilizing the Auto optimize feature.

## Supplementary information

Supplementary Info

## Data Availability

The data that support the findings of this study are available from the corresponding author upon reasonable request.

## References

[1] Kozel TR, Burnham-Marusich AR (2017). Point-of-Care Testing for Infectious Diseases: Past, Present, and Future. Journal of Clinical Microbiology.

[2] Shaw JLV (2016). Practical challenges related to point of care testing. Practical Laboratory Medicine.

[3] Islam MN (2017). RNA Biomarkers: Diagnostic and Prognostic Potentials and Recent Developments of Electrochemical Biosensors. Small Methods.

[4] Vaca L (2014). Point-of-care diagnostic tools to detect circulating microRNAS as biomarkers of disease. Sensors (Basel, Switzerland).

[5] Blackie EJ, Le R EC, Etchegoin PG (2009). Single-Molecule Surface-Enhanced Raman Spectroscopy of Nonresonant Molecules. Journal of the American Chemical Society.

[6] Aydin Ö, Altaş M, Kahraman M, Bayrak ÖF, Çulha M (2009). Differentiation of Healthy Brain Tissue and Tumors Using Surface-Enhanced Raman Scattering. Applied Spectroscopy.

[7] Ko H, Singamaneni S, Tsukruk VV (2008). Nanostructured Surfaces and Assemblies as SERS Media. Small.

[8] Chon H, Lee S, Son SW, Oh CH, Choo J (2009). Highly Sensitive Immunoassay of Lung Cancer Marker Carcinoembryonic Antigen Using Surface-Enhanced Raman Scattering of Hollow Gold Nanospheres. Analytical chemistry.

[9] Jahn, I. J., Radu, A. I., Weber, K., Cialla-May, D. & Popp, J. In *Nanotechnology Characterization Tools for Biosensing and Medical* Diagnosis (ed Challa S. S. R. Kumar) 1–66 (Springer Berlin Heidelberg, 2018).

[10] Doyle F (2017). Engineering Structurally Interacting RNA (sxRNA). Scientific reports.

[11] Clément J-E, Leray A, Bouhelier A, Finot E (2017). Spectral pointillism of enhanced Raman scattering for accessing structural and conformational information on single protein. Physical Chemistry Chemical Physics.

[12] Brulé T, Bouhelier A, Dereux A, Finot E (2016). Discrimination between Single Protein Conformations Using Dynamic SERS. ACS Sensors.

[13] Topală T, Bodoki A, Oprean L, Oprean R (2014). Bovine Serum Albumin Interactions with Metal Complexes. Clujul medical (1957).

[14] Bonnier F, Byrne HJ (2012). Understanding the molecular information contained in principal component analysis of vibrational spectra of biological systems. Analyst.

[15] Bosnick KA, Jiang J, Brus LE (2002). Fluctuations and Local Symmetry in Single-Molecule Rhodamine 6G Raman Scattering on Silver Nanocrystal Aggregates. The Journal of Physical Chemistry B.

[16] Zrimsek AB (2017). Single-Molecule Chemistry with Surface- and Tip-Enhanced Raman Spectroscopy. Chemical Reviews.

[17] Le R EC, Etchegoin PG (2012). Single-Molecule Surface-Enhanced Raman Spectroscopy. Annual Review of Physical Chemistry.

[18] Szeghalmi A, Kaminskyj S, Rösch P, Popp J, Gough KM (2007). Time Fluctuations and Imaging in the SERS Spectra of Fungal Hypha Grown on Nanostructured Substrates. The Journal of Physical Chemistry B.

[19] Taylor J, Huefner A, Li L, Wingfield J, Mahajan S (2016). Nanoparticles and intracellular applications of surface-enhanced Raman spectroscopy. Analyst.

[20] de Albuquerque CDL, Sobral-Filho RG, Poppi RJ, Brolo AG (2018). Digital Protocol for Chemical Analysis at Ultralow Concentrations by Surface-Enhanced Raman Scattering. Analytical chemistry.

[21] Singhal K, Kalkan AK (2010). Surface-Enhanced Raman Scattering Captures Conformational Changes of Single Photoactive Yellow Protein Molecules under Photoexcitation. Journal of the American Chemical Society.

[22] Tsai D-H (2011). Adsorption and Conformation of Serum Albumin Protein on Gold Nanoparticles Investigated Using Dimensional Measurements and *in Situ* Spectroscopic Methods. Langmuir: the ACS journal of surfaces and colloids.

[23] Van Regenmortel, M. H. V., Briand, J. P., Muller, S. & Plaué, S. In *Laboratory Techniques in Biochemistry and Molecular Biology* Vol. 19 (eds Burdon, R. H. & van Knippenberg, P. H.) 95–130 (Elsevier, 1988).

[24] Stewart S, Fredericks PM (1999). Surface-enhanced Raman spectroscopy of peptides and proteins adsorbed on an electrochemically prepared silver surface. Spectrochimica Acta Part A: Molecular and Biomolecular Spectroscopy.

[25] Podstawka E, Ozaki Y, Proniewicz LM (2005). Part III: Surface-Enhanced Raman Scattering of Amino Acids and Their Homodipeptide Monolayers Deposited onto Colloidal Gold Surface. Applied Spectroscopy.

[26] Madzharova F, Heiner Z, Kneipp J (2017). Surface Enhanced Hyper-Raman Scattering of the Amino Acids Tryptophan, Histidine, Phenylalanine, and Tyrosine. The Journal of Physical Chemistry C.

[27] Vargas-Obieta E (2016). Breast cancer detection based on serum sample surface enhanced Raman spectroscopy. Lasers in Medical Science.

[28] Kahraman M, Mullen ER, Korkmaz A, Wachsmann-Hogiu S (2017). Fundamentals and applications of SERS-based bioanalytical sensing. Nanophotonics.

[29] Xiaodan W (2018). Surface-enhanced Raman scattering investigation of bovine serum albumin by Au nanoparticles with different sizes. Journal of Applied Biomaterials & Functional Materials.

[30] Yang H, Zhang Y, Pöschl U (2010). Quantification of nitrotyrosine in nitrated proteins. Anal Bioanal Chem.

[31] Sanchez-Cortes S, Berenguel RM, Madejón A, Pérez-Méndez M (2002). Adsorption of Polyethyleneimine on Silver Nanoparticles and Its Interaction with a Plasmid DNA:  A Surface-Enhanced Raman Scattering Study. Biomacromolecules.

[32] Salerno M, Shayganpour A, Salis B, Dante S (2017). Surface-enhanced Raman scattering of self-assembled thiol monolayers and supported lipid membranes on thin anodic porous alumina. Beilstein journal of nanotechnology.

[33] Stranahan SM, Willets KA (2010). Super-resolution Optical Imaging of Single-Molecule SERS Hot Spots. Nano Letters.

[34] Pace CN, Vajdos F, Fee L, Grimsley G, Gray T (1995). How to measure and predict the molar absorption coefficient of a protein. Protein Science.

[35] Collier HB (1973). A note on the molar absorptivity of reduced Ellman’s reagent, 3-carboxylato-4-nitrothiophenolate. Analytical Biochemistry.

[36] Fowler JM, Stuart MC, Wong DKY (2007). Self-Assembled Layer of Thiolated Protein G as an Immunosensor Scaffold. Analytical chemistry.

[37] Sigg, C. D. & Buhmann, J. M. In *Proceedings of the 25th International Conference: Machine Learning*, 960–967 (2008).

