# Supplementary material for: Surface Enhanced Raman Spectroscopy for Single Molecule Protein Detection
Source: Sci Rep. 2019 Aug 26;9:12356. doi: 10.1038/s41598-019-48650-y (PMC6710251; doi:10.1038/s41598-019-48650-y)
Supplement: Supplementary file 1 — Supplementary Info [file 41598_2019_48650_MOESM1_ESM.pdf]

Supporting Information

Surface Enhanced Raman Spectroscopy for Single Molecule Protein Detection

Lamyaa M. Almehmadi,[1+] *Stephanie M. Curley,*[2+] *Natalya A. Tokranova,*[2] *Scott A. Tenenbaum*[2,3] *and Igor K. Lednev*[1*]

+co-first authors

[*]*Corresponding Author:*

Ilednev@albany.edu

[1]) Department of Chemistry, University at Albany, SUNY

1400 Washington Avenue, Albany NY 12222

[2]) College of Nanoscale Science and Engineering, SUNY Polytechnic Institute

257 Fuller Road, Albany, NY 12203

[3]) The RNA Institute, College of Arts and Science, University at Albany, SUNY,

1400 Washington Avenue, Albany NY 12222

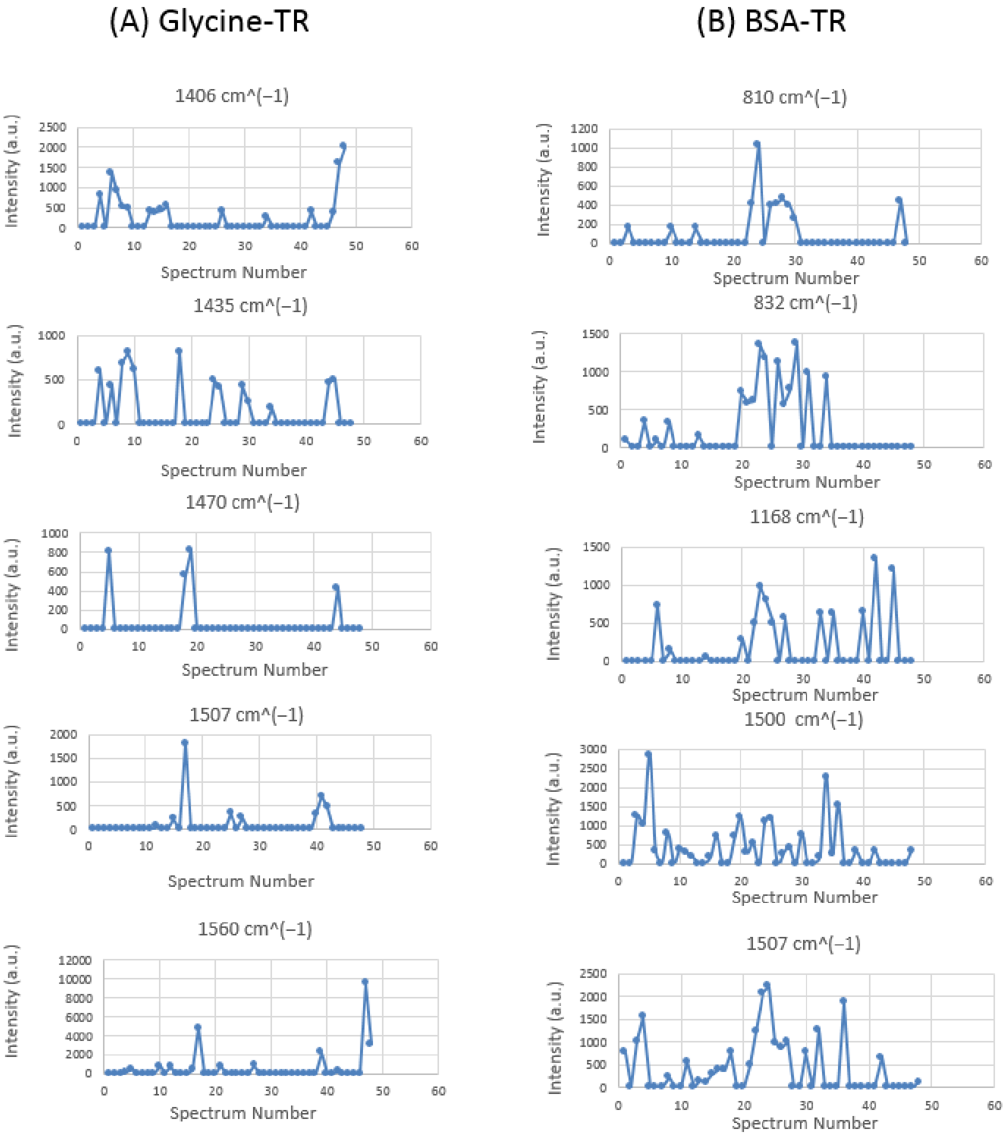

Figure S1. The intensity fluctuation of selected Raman peaks from the low loading (A) Glycine-TR and (B) BSA-TR spectra measured at individual spots on SERS substrates.

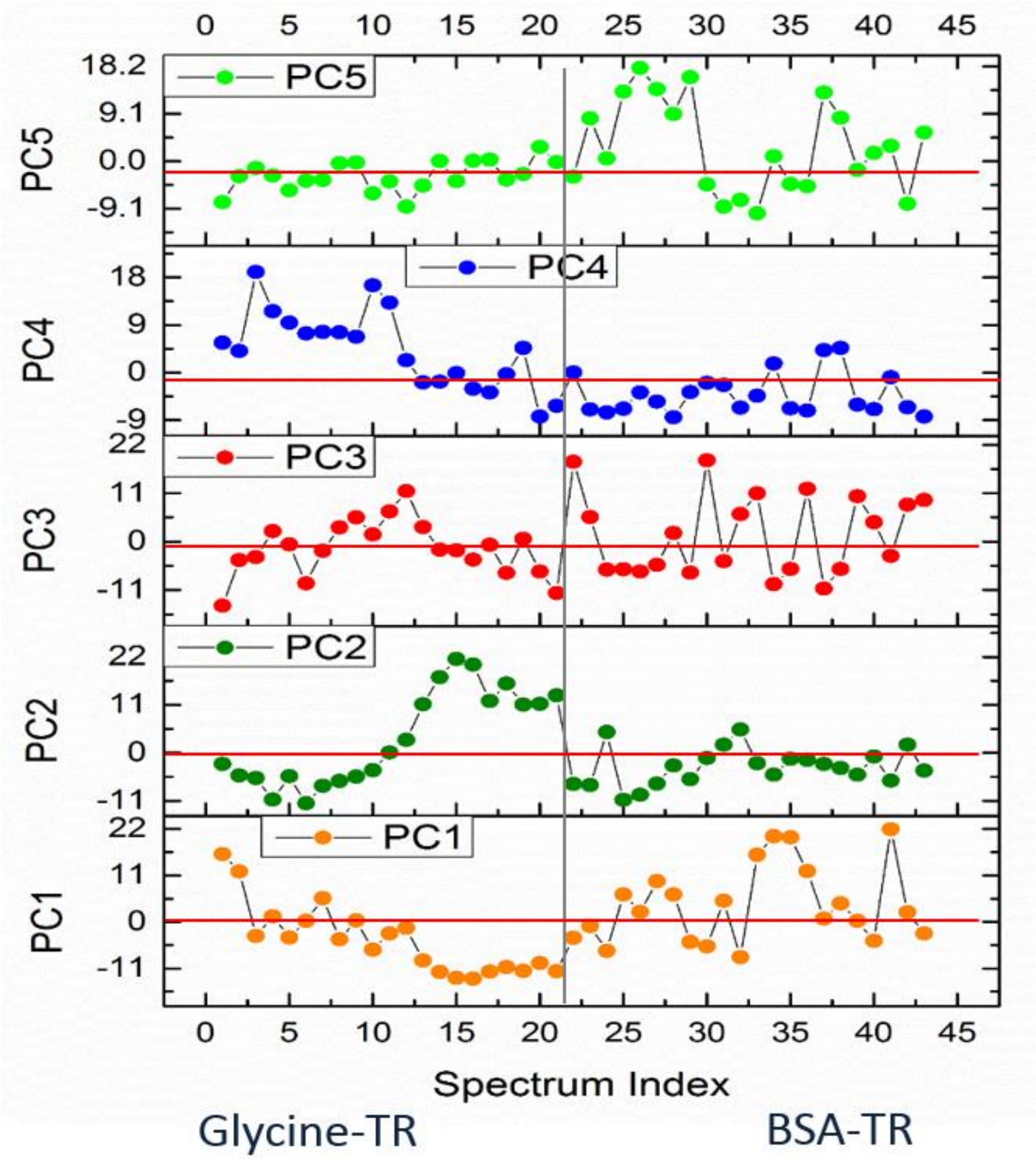

Figure S2. Associated PCs values within each spectrum of Glycine-TR and BSA-TR (spectrum index 1-22 and 23-43, respectively). The BSA-TR spectrum index in PC1 and PC2 shows almost an opposite trend to the Glycine-TR spectrum index. PC4 also shows similar trend to PC1 and PC2 where BSA-TR and Glycine-TR also have an opposite trend.

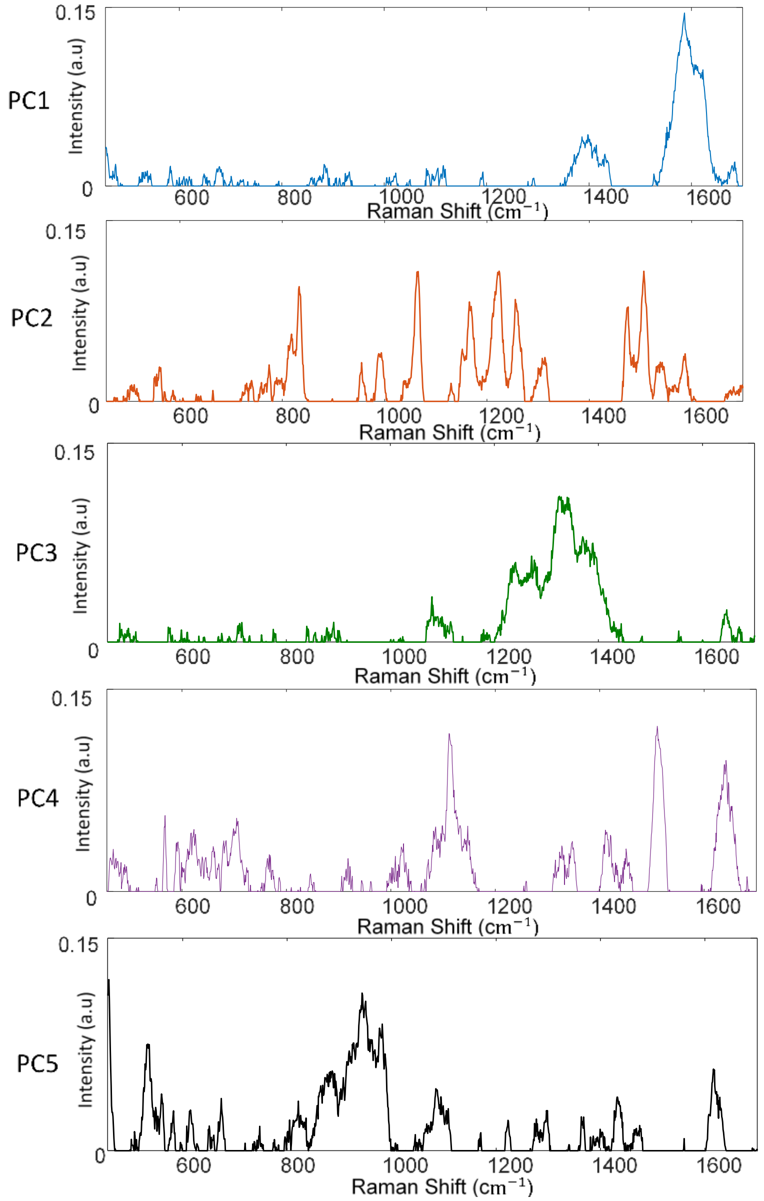

Figure S3. Positive loadings plot for PC1, PC2, PC3, PC4, and PC5.

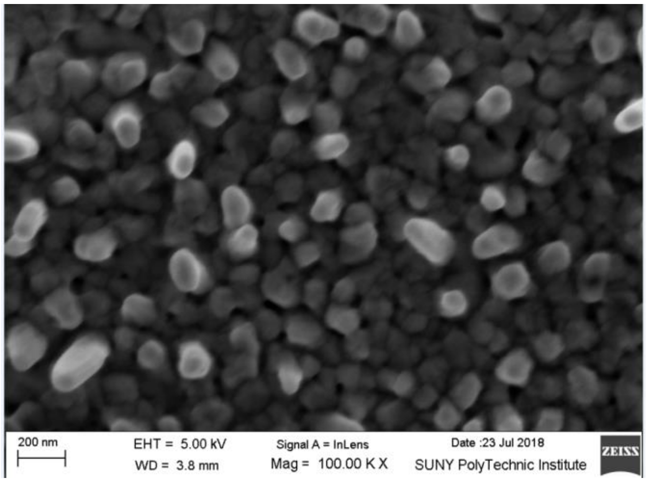

Figure S4. SEM image of the SERS substrate. The SEM image is taken by Scanning Electron Microscope (SEM) Zeiss LEO 1550.
